# 

**Published:** 1896-11-28

**Authors:** 


### EARLY EDITION.

**Notices of Births, Deaths, and Marriages** are inserted in this column at a charge of 2s. 6d. for an announcement not exceeding four lines.

### NOTICE TO CORRESPONDENTS.

All MS., Letters, Books for Review, and other matters intended for the Editor should be addressed THE EDITOR, "THE HOSPITAL," THE HOSPITAL BUILDING, 28 & 29, SOUTHAMPTON STREET, STRAND, W.C.

The Editor cannot undertake to return rejected MS., even when accompanied by stamped directed envelope.

**Notice to Advertisers and Subscribers.**

All Advertisements, Orders for Copies of THE HOSPITAL, and Business Communications should be addressed to **THE MANAGER, (Not to The Editor),** THE HOSPITAL, The Hospital Building, 28 & 29, Southampton Street, Strand, London, W.C.

The Cost of Subscription, post free, is as follows:—Per week, 2½d.; per quarter, 2s. 8d.; per half-year, 5s. 3d.; per annum, 10s. 6d. Foreign:—Per annum, 15s. 2d.; payable, in all cases, in advance.

**IMPORTANT NOTICE.**

The EDITORIAL and PUBLISHING OFFICES of "THE HOSPITAL" are now TRANSFERRED to their new premises, THE HOSPITAL BUILDING, 28 & 29, SOUTHAMPTON STREET, STRAND, LONDON, W.C.

**NOTICE.—Vol. XX. of "The Hospital" (April to September, 1896), in handsome cloth binding, is now on sale at the Office of this Journal, price 7s. 6d. Cases for binding the Numbers contained in this Volume 1s. 6d. Index to Vol. XX., 2½d., post free. The Monthly Part for December will be ready on December 1st, Price 6d., by post 8d.**

### Scraps and Gleanings.

The new Cancer Hospital at Glasgow was opened last month.

A cottage hospital is being built at Manly, New South Wales.

The Brighton Hospital Saturday Fund collected nearly £737 this year.

Last Hospital Sunday £954 was collected at Brighton, as against £883 in 1895.

During the coming year another block of the new building of the Sheffield Royal Hospital is to be undertaken.

A new cottage hospital—the Ellen Badger Cottage Hospital—has recently been opened at Shipston-on-Stour.

The proposal to build new offices for the Metropolitan Asylums Board on the Victoria Embankment has met with considerable opposition.

During the first six months of 1896 13,634 vessels were inspected by the officers of the Port of London Sanitary Committee.

A Moscow lady has recently given 515,000 roubles (nearly £55,000) to the University of Moscow to found a clinic with 25 free beds for diseases of the ear, throat, and nose.

As a result of the Hospital Saturday collections in Lincoln, Gainsborough, and Sleaford this year nearly £600 has been handed over to the Lincoln County Hospital.

Nearly £119 was collected this year in aid of the Sunderland and North Durham Eye Infirmary as the result of the annual house to house collection. £97 was obtained last year.

The annual report of the Dudley Dispensary states that 5,422 patients were treated during the past year, against 5,569 in the previous year. The average cost per head was 3s. 6½d. against 3s. 6½d.

A correspondent of the *Belfast Evening Telegraph* suggests that a hospital, to be known as the Victoria Memorial Hospital, should be erected as the Belfast memorial of the Queen's long reign.

A handsome ambulance brougham has been presented to the Turner Memorial Hospital, Keith, by the members of the St. John Ambulance classes who have been taught in Keith and the adjoining parishes.

The amount realised by the Coventry Hospital Saturday Working Men's Committee in 1896 was £1,336, or about £300 more than in 1895. The expenses were only £25, or rather under 2 per cent. of the collection.

The contractors for the Park Fever Hospital, now being built by the Metropolitan Asylums Board, have been offered a bonus of £3,100 if they can complete the hospital by July, 1897, instead of by September 7th, 1897.

In 1864 the death-rate of Edinburgh was 27 per 1,000. In 1894 it was 16·07. Owing to the measles epidemic in the first half of 1895 it rose to 19·17, falling again to 15·54 in the second half of 1895, and to 15·51 in the first half of this year.

A conference of representatives of sanitary authorities of the county of Chester held last month protested strongly against the proposed enforcement by the Chester County Council of the provision of isolation hospitals by local authorities throughout the county.

At the beginning of 1893 there were 37 dispensaries open in Lower and 47 in Upper Burmah. At the close of 1895 the numbers had risen to 43 and 50 respectively. Of these 83 provide beds for patients. The aggregate yearly number of patients rose from 503,002 in 1893 to 632,987 in 1895.

The Chairman of the Liverpool Port Sanitary and Hospitals Committee recently stated that for an estimated population of 650,000, and an additional 200,000 persons brought into the city in connection with the emigration trade, Liverpool had only about 600 beds for infectious diseases.

It is noticeable that the number of in-patients at the Burmese dispensaries is large only at the seaport towns and at the more important towns, such as Prome and Mandalay, in the interior. The Burmans, as a rule, are averse to indoor treatment, except when surgical measures are necessary.

The Sub-Committee of the Broken-Hill Hospital (New South Wales), appointed to arrange the details of a scheme for placing the hospital on a sound financial basis, has issued a circular explaining the proposal. It requests employers of all kinds of labour to collect subscriptions of not more than 1d. in the pound per week from employés, the employers subscribing an equal amount.

One of the earliest records of hospital treatment in Edinburgh tells of the leper house at Greenside, in the parish of Leith, built in 1520. In 1591 the inmates of this house were prohibited by the Town Council of Edinburgh from soliciting alms except by the clapper of one of their number, who alone was permitted to sit outside the door, the remainder being kept close prisoners; "and for the terrifying the said lepers to transgress the same" [it was deemed] "expedient that there be a gibbet set up at the gavell of the said hospital."

There were 257 hospitals and dispensaries (containing 3,073 beds) open in the Punjab at the end of the year 1895, an increase of 15 when compared with 1893; 56,172 in-patients were treated during 1895, as compared with 54,905 in 1893, and 43,770 in 1887. The daily average number under treatment was 1,859, consisting approximately of 1,407 men, 331 women, and 121 children. The number of out-patients was 3,204,445, compared with 3,061,918 in 1893. The total expenditure amounted to Rs.5,37,399 in 1895, against Rs.5,30,959 in 1893.

### The Student of Medicine.

### APPOINTMENTS.

E. Bark, M.R.C.S., L.R.C.P.Lond., appointed Consulting Surgeon to the Birmingham Lying-in Charity. W. B. Bell, M.R.C.S., L.R.C.P., appointed Honorary Physician to the New Brighton Convalescent Home for Women and Children, and Honorary Assistant Physician to the Liverpool Home for Aged Mariners. W. H. Coates, M.A., M.B.Durh., appointed Medical Officer of Health for the Patrington Rural District, and Medical Officer to the Workhouse of the Patrington Union. Dr. W. Davidson, appointed Medical Officer for the Gringley District of the Retford Union. A. E. Giles, M.D.Lond., B.Sc., F.R.C.S.Edin., appointed House Surgeon to the Out-patients at the Chelsea Hospital for Women. L. Grant, M.D.Edin., appointed Resident Medical Officer to the Gesto Hospital, Isle of Skye. A. E. Griffin, M.A.Cantab., M.R.C.S.Eng., L.R.C.P., Lond., appointed House Physician to the Hospital for Consumption and Diseases of the Chest, Brompton, S.W. W. Harris, M.D., M.R.C.P., appointed Senior House Physician to the National Hospital for the Paralysed and Epileptic, Queen Square. R. C. Jameson, M.B., C.M.Edin., appointed Dispensary Surgeon to the Bradford Infirmary. T. J. Kennedy, L.R.C.P., L.R.C.S.I., appointed Medical Officer to the Toomevara Dispensary. B. H. Lees, M.A., M.B.Camb., appointed Medical Officer for the Second B. District of the East Preston Union. P. Macdonald, M.A., M.B., C.M.Aberd., appointed Dispensary Surgeon to the Bradford Infirmary. Dr. G. P. O'Connor, appointed Medical Officer for the Fourth District (Manea) of the North Witchford Union. J. O'Dwyer, L.R.C.S. L.R.C.P.I. appointed Medical Officer to the Tipperary Dispensary District. A. L. Salmon, M.R.C.S., L.S.A., appointed Medical Officer of the Workhouse and of the St. Clement District of the Truro Union. A. E. Scott, M.R.C.S., L.R.C.P., appointed Assistant Resident Medical Officer to the North-West London Hospital, N.W. W. Shackleton, M.B., B.Ch., B.A.O.Trin.Coll., L.M.Rotunda Hosp.Dub., appointed House Surgeon to the Wolverhampton Eye Infirmary. R. Slocock, B.A.Oxford, M.R.C.S., L.R.C.P., appointed Assistant House Surgeon to the Royal Portsmouth Hospital. Dr. W. A. Stevenson, appointed Medical Officer for the Helvertoft District of the Rugby Union. J. P. Stewart, M.A., M.B., C.M.Edin., M.R.C.S.Eng., L.R.C.P.Lond., appointed Junior House Physician to the National Hospital for the Paralysed and Epileptic, Queen Square, Bloomsbury, W.C. E. R. C. Swainson, M.A., M.B., B.C.Cantab., M.R.C.S., L.R.C.P., appointed Resident Medical Officer to the North-West London Hospital, N.W. E. G. Walls, M.R.C.S., L.R.C.P.Lond., appointed Medical Officer for the Reevesby District of the Horncastle Union. Dr. W. R. White, appointed Medical Officer for the Benenden District of the Cranbrook Union.

St. Thomas's Hospital House Appointments.—The following gentlemen have been selected as house officers from Tuesday, December 1st, 1896: House Physicians: A. W. Sikes, B.Sc.Lond., L.R.C.P., M.R.C.S., J. P. Scatchard, L.R.C.P., M.R.C.S., G. J. Conford, B.A., M.B., B.Ch.Oxon, L.R.C.P., M.R.C.S. (extension), L. W. Richards, M.B., B.S.Durh., L.R.C.P., M.R.C.S. (extension). House Surgeons: B. Dyball, L.R.C.P., M.R.C.S. (extension), P. W. Kent, L.R.C.P., M.R.C.S. (extension), J. Smith, M.A., M.B., B.C.Cantab., L.R.C.P., M.R.C.S. (extension), W. D. Frazer, L.R.C.P., M.R.C.S. (extension). Assistant House Surgeons: A. J. Martineau, L.R.C.P., M.R.C.S. (exten-

### THE STUDENTS OF MEDICINE—*continued.*

sion), F. H. Gervis, L.R.C.P., M.R.C.S. (extension), R. G. Strange, L.R.C.P., M.R.C.S. (extension), G. E. O. Taylor, L.R.C.P., M.R.C.S. (extension). Obstetric House Physicians: Senior, E. L. Collis, B.A., M.B., B.Ch.Oxon, L.R.C.P., M.R.C.S., and junior, A. L. Home, L.R.C.P., M.R.C.S. Clinical Assistants in the Special Department for Diseases of the Throat: H. G. Toombs, L.R.C.P., M.R.C.S. (extension), W. J. Orbell Ray, L.R.C.P., M.R.C.S. (extension). Clinical Assistants in the Special Departments for Diseases of the Skin: A. H. P. Dawnay, L.R.C.P., M.R.C.S. (extension), R. Lawson, L.R.C.P., M.R.C.S. (extension). Clinical Assistants in the Special Department for Diseases of the Ear: E. H. T. Nash, L.R.C.P., M.R.C.S., E. Hopkinson, L.R.C.P., M.R.C.S. Clinical Assistants in the Electrical Department: C. G. Seligmann, L.R.C.P., M.R.C.S., E. Stainer, M.A., M.B., B.Ch.Oxon.

### PASS LISTS.

#### University of London.

M.B. Examination.

First Division.—C. Bolton, B.Sc., University College; T. R. H. Bucknall, University College; M. W. Coleman, St. Bartholomew's Hospital; P. R. Cooper, B.Sc., Owens College and Manchester Royal Infirmary: R. W. Dedgson, St. Mary's Hospital; C. H. Drake, St. Bartholomew's Hospital; E. G. D. Drury, St. Bartholomew's Hospital; W. d'E. Emery, B.Sc., St. Bartholomew's and Queen's Hospital, Birmingham; A. B. Jones, University College; J. D. Russell, University College; A. W. Sanders, St. Mary's Hospital; W. B. L. Trotter, University College; W. C. Wood, St. Mary's Hospital.

Second Division.—E. G. B. Adams, St. Bartholomew's Hospital; W. Allport, Queen's College and General Hospital, Birmingham; F. A. Arnold, London Hospital: E. D. Aubin, Middlesex Hospital; C. Banting, University College; J. S. Boden, King's College; W. F. V. Bonney, Middlesex Hospital; E. B. Bostock, Mason College and General Hospital, Birmingham; A. Bousfield, B.Sc., King's College; P. W. Brigstocke, St. Bartholomew's Hospital; W. R. Bryett, B.A., King's College; A. G. Butler, Guy's Hospital; W. E. Dixon, B.Sc., St. Thomas's Hospital; A. R. J. Douglas, St. Bartholomew's Hospital; G. R. Elwin, St. Mary's Hospital; W. H. Holliday, Yorkshire College; A. Howell, St. Mary's Hospital; S. P. Huggins, St. Bartholomew's Hospital; J. Hussey, St. Bartholomew's Hospital; H. W. Jackson, University College; C. E. M. Kelly, Owens College; A. D. Ketchen, Glasgow University and Western Infirmary; C. H. Langford, St. Bartholomew's Hospital; Octavia Margaret S. Lewin, London School of Medicine and Royal Free Hospital; A. Marriott, University College and Sheffield Medical School; W. G. Mortimer, London Hospital; Winifred Secretan Patch, B.Sc., London School of Medicine for Women; N. H. Pike, Guy's Hospital; E. Pratt, St. Bartholomew's Hospital; G. B. Price, Bristol Medical School and St. Bartholomew's Hospital; C. Roberts, Middlesex Hospital; W. G. Savage, B.Sc., University College; E. T. Scowby, Guy's Hospital; A. H. Spicer, Guy's Hospital; E. I. Spriggs, Guy's Hospital; H. E. Thompson, St. Bartholomew's Hospital; A. Thorne, St. Mary's Hospital; F. B. Thornton, St. Thomas's Hospital; E. O. Thurston, St. Thomas's Hospital; A. B. Tucker, St. Bartholomew's Hospital; Ethel M. Vaughan, London School of Medicine and Royal Free Hospital; K. B. J. Vickers, St. Thomas's Hospital; Mabel E. Webb, London School of Medicine for Women; Sarah E. White, B.Sc., London School of Medicine and Royal Free Hospital; R. P. Williams, King's College; W. Wrangham, St. Bartholomew's Hospital.

#### Conjoint Board in Ireland.

Second Professional Examination.

The following candidates have passed this examination as undernoted:

Completed the Examination.—C. W. Crowe, T. O'C. Donelan, M. F. Hession, M. J. Hynes, C. G. Jones, G. Kennedy, N. P. Kirby, J. W. Langstaff, J. F. Loughrey, H. S. Maxwell, J. I. M'Grath, H. J. C. Wallace, A. A. Woods.

Passed in Anatomy.—W. J. Anglim, A. D. Dunwoody, R. J. Franklin, P. A. Frazer, J. L. Jones, L. G. de Rosario, T. A. E. Rooke, G. F. Sheehan, J. Whelan.

Passed in Physiology.—W. J. Anglim, W. R. Blackwell, G. C. L. Kerans.

Passed in Materia Medica.—R. B. Daly, P. J. M'Ginn, L. G. de Rosario, M. J. Russell, R. J. White.

Passed in Histology.—W. J. Anglim, A. D. C. Cummins, R. B. Daly, P. A. Frazer, C. A. A. Lener, T. A. E. Rooke, G. Tickell.

#### Triple Qualification Examination in Edinburgh.

Third Examination (5 years' course).—Of 10 candidates entered, the following five passed the examination: H. S. Anderson, County Antrim; E. F. L. de Jersey, Hampshire; J. Dick, Greenock; C. C. Murison, India; and Rosalie Berthon, London. Three candidates passed in one or two divisions.

Final Examination.—Of 112 candidates entered, the following 41 passed the examination, and were admitted L.R.C.P.E., L.R.C.S.E., and L.F.P.S.G.: A. N. Davies, Warwickshire; R. S. Williams, Liverpool; D. L. Spillane, Listowel; H. H. Busteed, Madras; E. H. Birchall, Rainhill; M. Hall, Wigan; E. Dobbs, Sheffield: C. W. Holmested, Essex; Janet Gertrude Horwood, Aylesbury; E. Frost, Ashton-under-Lyne; J. Dunlop, Glasgow; A. J. Grant, Canada; J. Roche, County Cork; W. H. Irwin, Leeds; Emily Frances Chestnut FitzSimon, Tralee; T. Macdowell, County Down; A. B. Hood, Glasgow; H. Exell, Kent; M. Higgins, County Derry; C. H. Thackrah, Halifax; A. G. Hays, West Bromwich; J. C. Hamilton, Ireland; J. F. M'Fadyen, Scotland; F. G. Henderson, India; W. Scot, India; B. G. Roscoe, Liverpool; E. Wright, Dublin; S. G.A. Jeffs, Great Grimsby; J. J. W. Campbell, Surrey; F. E. Bissell, Birmingham; J. Kennedy, County Antrim; W. Latham, Hindley, S. B. Gay, Swindon; J. R. Hatfield, Dewsbury; Louise Blanch Smith, Calcutta; J. A. Preston, Ireland, ; M. A. Menon, India; H. W. J. J. G. Cattell, Mauritius; J. L. Brownridge, Paisley; M. L. Neylon, Australia; and E. Blair, South Shields. In addition to the above 11 candidates passed in medicine, 5 in surgery, 14 in midwifery, and 11 in medical jurisprudence.

